# Risk Factors for Transient Hypoparathyroidism after Total Thyroidectomy: Insights from a Cohort Analysis

**DOI:** 10.3390/jcm13113326

**Published:** 2024-06-05

**Authors:** Giuseppa Graceffa, Antonella Lopes, Giuseppina Orlando, Sergio Mazzola, Fabrizio Vassallo, Francesco Curione, Pierina Richiusa, Stefano Radellini, Giuseppina Melfa, Gregorio Scerrino

**Affiliations:** 1 Unit of General and Oncology Surgery, Department of Surgical Oncological and Oral Sciences, Policlinico “P. Giaccone”, University of Palermo, Via Liborio Giuffré 5, 90127 Palermo, Italy; giuseppa.graceffa@unipa.it (G.G.); antonella.lopes22@gmail.com (A.L.); 2 Unit of General and Emergency Surgery, Department of Surgical Oncological and Oral Sciences, Policlinico “P. Giaccone”, University of Palermo, Via Liborio Giuffré 5, 90127 Palermo, Italy; fabris.fv@gmail.com (F.V.); curionef@gmail.com (F.C.); irene_melfa@yahoo.it (G.M.); 3 Unit of Clinical Epidemiology and Tumor Registry, Department of Laboratory Diagnostics, Policlinico “P. Giaccone”, University of Palermo, Via Liborio Giuffré 5, 90127 Palermo, Italy; mazzolasergio3@gmail.com; 4 Department of Health Promotion Sciences Maternal and Infantile Care, Internal Medicine and Medical Specialties (PROMISE), Section of Endocrinology, University of Palermo, Via Liborio Giuffré 5, 90127 Palermo, Italy; pierina.richiusa@policlinico.pa.it (P.R.); stefano.radellini@policlinico.pa.it (S.R.); 5 Unit of Endocrine Surgery, Department of Surgical Oncological and Oral Sciences, Policlinico “P. Giaccone”, University of Palermo, Via Liborio Giuffré 5, 90127 Palermo, Italy; gregorio.scerrino@tiscali.it

**Keywords:** transient hypoparathyroidism, thyroidectomy outcomes, surgical complications, incidental parathyroidectomy, vitamin D, surgeon experience

## Abstract

**Background:** Transient hypoparathyroidism (TH) is the main post-thyroidectomy complication, significantly impacting surgical outcomes, hospitalization length, and perceived perceived quality of life understood as mental and physical well-being. This study aims to identify possible associated risk factors. **Methods:** We analyzed 238 thyroidectomies (2020–2022), excluding instances of partial surgery, primary hyperparathyroidism, neck irradiation history, and renal failure. The variables considered were as follows: demographics, histology, autoimmunity, thyroid function, pre- and postoperative Vitamin D levels (where available), type of surgery, number of incidentally removed parathyroid glands (IRP), and surgeons’ experience (>1000 thyroidectomies, <500, in training). Univariate analysis applied: χ^2^, Fisher’s exact test for categorical variables, and Student’s *t*-test for continuous variables. Subsequently, logistic multivariate analysis with stepwise selection was performed. **Results:** Univariate analysis did not yield statistically significant results for the considered variables. The ‘No Complications’ group displayed a mean age of 55 years, whereas the TH group showed a mean age of 51 (*p*-value = 0.055). We considered this result to be marginally significant. Subsequently, we constructed a multivariate logistic model. This model (AIC = 245.02) indicated that the absence of incidental parathyroidectomy was associated with the age class >55 years, presenting an odds ratio (OR) of 9.015 (*p*-value < 0.05). Simultaneously, the age class >55 years exhibited protective effects against TH, demonstrating an OR of 0.085 (*p*-value < 0.01). Similarly, the absence of incidental parathyroidectomy was found to be protective against TH, with an OR of 0.208 (*p*-value < 0.01). **Conclusions:** Multivariate analysis highlighted that having “No IRP” was protective against TH, while younger age was a risk factor. Surgeon experience does not seem to correlate with IRP or outcomes, assuming there is adequate tutoring and a case volume close to 500 to ensure good results. The effect of reimplantation has not been evident in transient hypoparathyroidism.

## 1. Introduction

Among the complications of total thyroidectomy, hypoparathyroidism is the most frequent [1]. The definition of “hypoparathyroidism” following thyroid surgery is extremely variable across different sources. The use of the “calcemia” parameter is the most commonly accepted. In this context, calcium values < 8 mg/100 mL (<2 mmol/L) correspond to the most widespread definition. Employing this criterion, the frequency of hypoparathyroidism appears to be very high (even more than 50% of total thyroidectomies) [1,2]. If the calcemia cut-off is reduced to 7.6 mg/100 mL, the frequency of this complication drops to less than 30% [3]. Nonetheless, hypoparathyroidism is a common condition following bilateral thyroidectomy. It can result from the removal of parathyroid tissue, or from damage to or devascularization of some parathyroid glands [4].

Current consensus on predicting hypocalcemia risk is lacking. Although retrospective studies suggest that postoperative intact Parathyroid Hormone (iPTH) may foresee hypocalcemia, optimal cut-off values and timing for iPTH evaluation vary. Yet, this could be a valuable indicator for hypocalcemia in susceptible patients [5,6,7].

Hypoparathyroidism can be temporary (generally resolving within 6 months post-surgery) or permanent. It may require variable supplementation of calcium and vitamin D. In any case, it is a condition that, even in its more transient and less severe forms, can prolong hospitalization after thyroidectomy, while in severe cases, it can lead to serious complications [8,9].

The aim of this study is to assess the factors associated with this complication in a cohort of patients undergoing total thyroidectomy at a tertiary center.

## 2. Materials and Methods

This retrospective cohort study was performed at our institution (Department of Surgical, Oncological, and Stomatological Disciplines at the University of Palermo). The subjects included in the study consisted of all patients who underwent total thyroidectomy from January 2020 to December 2022, for whom complete pre-, intra-, and postoperative data were available, along with an updated follow-up as of December 2023. We excluded partial thyroidectomy (lobectomy and/or isthmusectomy), coexistence of hyperparathyroidism, preoperative therapy with calcium and vitamin D, and all reoperations (either early, meaning within the same hospitalization, or later on). All thyroidectomy patients were initially treated with L-thyroxine at a dose of 1.7 μg/kg of body weight. Subsequent therapy adjustments aimed to achieve an ideal TSH level of 0.5–1 mU/L, with a tolerance range of 0.4 to 4 mU/L. Patients with lower levels (due to a suppressive therapy chosen by the referring endocrinologist, 3 patients) were excluded because of the known effect of hyperthyroidism on calcium–phosphorus metabolism. Higher TSH levels prompted an investigation into the possible persistence of thyroid tissue in the neck. Patients with hyperthyroidism, particularly those with Graves’ Disease, with residual tissue maintaining a high antibody pattern, were not included in the study (2 patients). We also excluded 8 patients who had undergone parathyroid reimplantation during thyroidectomy. Patients with a history of neck irradiation and renal failure were also considered for exclusion. More specifically, only patients classified as ‘low’ or ‘moderate’ risk according to the KDIGO guidelines [10] were included. Patients with intestinal malabsorption (previous resection, known inflammatory bowel diseases, celiac disease) were excluded from the study. We also excluded one patient who developed a neck hematoma requiring early re-exploration (3 h after the thyroidectomy). Finally, patients with cancer other than papillary were also excluded. The choice of this cohort was aimed to exclude any condition that could influence calcium metabolism or expose the parathyroids to an iatrogenic risk higher than the standard of thyroidectomy.

As usual, in our department, all patients undergoing thyroid surgery had their vitamin D levels corrected as needed, and all patients underwent surgery with levels between 25 and 40 ng/mL.

The goal of our management of thyroidectomy patients is a hospital stay of more than 24 h but not exceeding 48 h. Any further extension of the hospital stay for the optimization of calcium levels, in accordance with the established criteria, was considered as a criterion for defining transient hypoparathyroidism.

Regarding the variables considered, we included distinct groups of patients suffering from benign disease (multinodular goiter), malignant pathology (primarily papillary carcinomas), and Graves’ disease. This category encompassed diffuse toxic goiters with clinical signs of hyperthyroidism, positive antibody patterns, and TRAb positivity (>1.8 IU/L).

In our cohort, patients with Graves’ Disease underwent thyroidectomy within a variable time frame ranging from 12 to 36 months.

Autoimmunity was deemed positive in the presence of positivity of anti-TPO antibodies and after histological confirmation. Thyroid dysfunctions (hyperthyroidism and hypothyroidism), if present, were normalized; in the former case, this was performed with synthetic antithyroid drugs (Methimazole or Propylthiouracil), and in the latter with replacement therapy (L-thyroxine). Hyperthyroid subjects were considered eligible for surgery if FT3 values were within the normal range, although TSH often remained suppressed especially in several patients with Grave’s disease. Unilateral central neck dissection (CND) was performed solely for prophylactic purposes, in the absence of macroscopic evidence of enlarged central lymph nodes, with papillary carcinomas confirmed cytologically at a size corresponding to T2. Bilateral CND was applied in T3 cases. The number of parathyroids removed was assessed at the time of the definitive histological examination. Lastly, surgeons were classified based on the actual scenario in the department where the study was conducted into three levels of expertise: (A) a volume of activity of 1000 or more thyroidectomies, at least 15 years of activity (2 operators); (B) more than 300 thyroidectomies over 7 years of activity (1 operator); (C) a learning curve considered not yet completed (less than 100 thyroidectomies, 1 operator, or residents, 4 operators). The third group performed the surgery under variable intensity tutoring by one of the surgeons from the first group.

To pinpoint every patient affected by post-surgical hypoparathyroidism, individuals were considered for inclusion if they satisfied the following three criteria the day subsequent to their surgery:

(1) Adjusted calcium levels < 8 mg%;
(2) PTH levels ≤ 10 pg/mL;
(3) Requirement for calcium and/or activated vitamin D supplementation due to the emergence of symptoms indicative of hypocalcemia.

Calcium levels below the normal range, when accompanied by PTH values ≥ 15 pg/mL, typically do not present any symptoms and do not necessitate supplementation, as also reported in the literature [11,12].

Patients with PTH levels between 10 and 15 pg/mL who were hypocalcemic had an additional postoperative day of hospital stay, although no calcium and vitamin D supplementation was administered.

We considered all subjects as recovered who returned to normal ranges of serum calcium and PTH levels, specifically PTH ≥ 15 pg/mL and serum calcium ≥ 8.4 mg%.

The current research adhered to the principles outlined in the Declaration of Helsinki (1964) and its subsequent modifications. Participants provided informed consent; no approval from the Institutional Ethics Committee was requested since the study was based on the simple observation of anonymous data entered into a dataset and did not involve any change in treatment for the included patients.

### Statistical Analysis

In the initial phase, a univariate analysis was conducted applying the Chi-square (χ^2^) test and/or Fisher’s exact test for categorical variables, and Student’s *t*-test for continuous variables. Subsequently, a logistic multivariate analysis was carried out to examine whether age, which showed a marginally significant association with postoperative hypoparathyroidism, could be linked with any of the following variables: gender, disease (categorized into: multinodular goiter, carcinoma, Graves’ disease), autoimmunity, thyroid function (euthyroidism, hyperthyroidism, hypothyroidism), type of surgery (thyroidectomy, thyroidectomy and central neck dissection, thyroidectomy and bilateral central neck dissection), and the number of parathyroid glands accidentally removed. This was carried out using the stepwise method to identify the best model based on the significance of the regression coefficients and the lowest Akaike Information Criterion (AIC) value.

Statistical analyses were performed using RStudio software (version 3.4.1 of 30 June 2017) for R (version 2.1).

## 3. Results

In the cohort under consideration, 52 out of 238 patients (21.85%) had transient hypocalcemia (TH).

When evaluating the group of patients without transient hypoparathyroidism separately from those who developed this complication, it is observed that risk factors such as hyperthyroidism (whose incidence increases from 22% to 25% when moving from the uncomplicated group to those with transient hypoparathyroidism) and, even more so, Graves’ disease (from 12% to 19%, respectively) show a clear relative increase. Meanwhile, other hypothesized risk factors related to thyroid disease have demonstrated behaviors different to the expected outcomes (Table 1).

Hyperthyroidism, and even more so Graves’ disease, exhibits a relatively increased representation in cases of hypoparathyroidism compared to the group where such complications did not occur.

In contrast, when comparing the two groups on variables represented by risk factors related to surgery (number of parathyroid glands identified, surgeon’s experience), only the first variable was found to be proportionally more represented in the group of patients with transient hypoparathyroidism, while no significant variation was observed in the latter (Table 2).

The only variable that clearly changes in the group of patients with post-surgical hypoparathyroidism is the accidental removal of parathyroid tissue.

Overall, 11 patients (4.6%) developed permanent hypoparathyroidism.

Among the 8 patients who underwent parathyroid reimplantation within the sternocleidomastoid muscle bundles, 2 (25%) developed transient hypoparathyroidism, which resolved between 3 and 8 weeks postoperatively. This reimplantation was due to the evident devascularization of one parathyroid gland, or by its accidental removal during the thyroidectomy maneuvers. The group of patients who underwent parathyroid reimplantation, compared to the group in which this procedure was excluded, showed a non-significant difference (*p* = 0.68).

Univariate analysis of the overall data on the variables considered did not reach statistical significance (Table 3). However, it was noted that the “No TH” group had an average age of 55 years, whereas the “TH” group had an average age of 51 years; furthermore, the difference between the two ages showed “marginal” significance with a *p*-value = 0.055. Given this difference, and the seemingly unbalanced percentage between the “IRP” and “no-IRP” groups in terms of TH, we proceeded with a multivariate logistic analysis using the stepwise method.

The results of the model applied, deemed reliable with an AIC = 245.02, are presented in Table 4. Analyzing this table reveals that IRP = 0 is correlated with the age group > 55 years with an odds ratio (OR) of 9.015 (*p*-value < 0.05); moreover, the age group > 55 years appears to be protective against TH with an OR = 0.085 (*p*-value < 0.01), as is IRP = 0 regarding TH with an OR = 0.208 (*p*-value < 0.01).

The multivariate analysis thus indicates that IRP = 0 is a protective condition against TH, while conversely, patients undergoing surgical intervention under the age of 55 years are at risk.

In our cohort, the recovery to normal conditions of calcium and PTH occurred within a time range of 2 to 11 weeks. While recovery in longer periods is possible, in practice, the 11 patients who exceeded this limit developed permanent hypoparathyroidism. The follow-up for all patients included in the present study extended beyond 12 months.

## 4. Discussion

Definitive post-thyroidectomy hypoparathyroidism is considered as an evolution of the transient form [1,3,4,6]. Here, we have intentionally omitted this potential (and certainly possible) evolution calculating only its incidence, which was too low (11 cases) to perform statistical evaluations. Definitive hypoparathyroidism is associated with severe complications (cataract, kidney stones) which, along with the persistence of hypocalcemia symptoms, result in a substantial impairment of quality of life [13,14].

Transient hypoparathyroidism is the most frequent cause of delayed discharge after total thyroidectomy, whereas discharge is usually accepted even after 24 h in the absence of complications [7,15,16].

The accidental removal of parathyroid tissue is one of the main risk factors for post-thyroidectomy hypocalcemia. In the extensive series of Melot and coll. (over 1500 patients divided into two groups based on the presence or absence of IRP), a significantly higher prevalence of first-day post-operative hypocalcemia (17.1% versus 9.6%, *p* < 0.0001), transient hypocalcemia (17.9% versus 11.5%, *p* = 0.006), and permanent hypoparathyroidism (4.7% versus 1.6%, *p* = 0.002) was observed. Moreover, the number of parathyroids removed substantially affects the risk of developing permanent hypoparathyroidism (13.3% with 2 IRP) [14]. In turn, IRP was more common in surgery for malignancy compared to benign pathology and, as expected, in central neck dissections compared to simple thyroidectomy [17].

The number of IRPs varies in frequency, and in one study incidental parathyroidectomy was found in 24.9% of total thyroidectomies [18]. However, only a few studies have found an association with hypocalcemia [19,20], while in some cases, this association has been shown to be only biochemical, but not clinical [21]. In turn, the intrathyroidal position of an IRP is the cause in over 40% of cases [18].

Privitera and coll [22] analyzed the incidence and risk factors for postoperative hypoparathyroidism. Out of 734 patients, 230 (31.3%) exhibited a postoperative PTH level of <12 pg/mL. Significant risk factors for postoperative hypoparathyroidism were identified as female gender, age below 40, neck dissection, lymph node dissection yield, and IRP, which occurred in 16.6% of cases. 

In regard to the relationship between hypoparathyroidism and neck dissection, we did not find significant differences. However, it should be emphasized that in the cohort considered, only 19 patients underwent this additional procedure, and of these, only 7 underwent a bilateral operation.

Paladino and Coll [23] focused on the incidence of IRP and its impact on postoperative calcemia, examining patients undergoing total thyroidectomy from February 2016 to May 2018. Among the 766 patients examined, parathyroid tissue was incidentally included in the thyroid specimens for 40 patients (5.2%): 30 cases were IRP, and 10 were fragments. Postoperative hypocalcemia was observed in 97 patients (12.6%), with 90 experiencing transient and 5 enduring definitive hypoparathyroidism. Among those with IRP, 13 out of 30 (43.3%) encountered transient hypoparathyroidism.

This study underscores total thyroidectomy and intrathyroid localization of parathyroid glands as significant risk factors for IRP and postoperative hypocalcemia.

Although the univariate analysis did not reveal clear significances for the variables examined, the application of multivariate analysis, developed from the variable that was marginally significant (age), allowed us to determine that patients older than 55 years tended to be protected against hypocalcemia. It could be hypothesized that hormonal changes play a role in this difference. Specific receptors for female hormones and cortisol have indeed been identified in parathyroid cells [24]. Given that the patient cohort examined is predominantly female, the result could be related to the decline of such hormones in older ages, which could in turn lead to some receptor changes capable of making the parathyroids of older women, similarly to those of men, more reactive to surgical trauma and thus better adapted to a rapid functional recovery [25]. However, these are merely hypotheses that cannot be directly proven with the currently available data, although the correlation between age and TH, after thyroidectomy as well as parathyroidectomy, has been repeatedly demonstrated [22,26,27].

As showed in several papers previously published [17,18,19,20,21,22,23], the absence of “IRP” proved to be protective against TH at the multivariate analysis also in the present study.

Another interesting finding pertains to the association between the surgeon’s experience and the onset of hypoparathyroidism. In this respect, the results warrant careful examination. Although specific analyses on patient subgroups were not conducted, it can be hypothesized that the more ‘challenging’ cases (e.g., due to gland volume, presence of thyroiditis, more difficult anatomical conditions, necessity of lymph node dissection) were managed by senior surgeons, while it is assumed that residents handled more elementary situations. This might explain, on one hand, the even better outcomes of the mid-experienced surgeon compared to the seniors and, conversely, the relative closeness of the residents’ results. The literature data on this matter are somewhat conflicting, although there is a general trend suggesting a linear association between overall complications and the surgeon’s volume. It should be noted that the ‘safety volume’ is not always clearly established [28] or is set according to extremely variable criteria [29,30]. Regarding the specific context of residents, a recent meta-analysis tends to show a minimal negative impact of their participation in thyroidectomy procedures if they are adequately supported [31]. This latter result appears to be quite consistent with our observations. However, it is important to clarify a difference in our method of categorizing the surgeons’ expertise, which is based on the total volume of procedures rather than the annual volume of thyroidectomies.

Overall, the findings of our study tend to highlight, in determining the risk of transient post-thyroidectomy hypoparathyroidism, a predominance of biological factors, centered primarily on age, rather than on aspects of surgical technique. The accidental removal of parathyroid tissue might also be more closely related to an anomalous position of the gland (subcapsular or intrathyroidal) rather than to other factors more directly associated with imperfections in surgical technique. Accepting this thesis, the emphasis placed on linking this adverse event to the surgeon’s experience seems to diminish, assuming that the technical standards concerning the identification and “in situ” preservation of the parathyroid glands are observed.

However, we are aware that there is a substantial body of literature supporting the significant relevance of surgeon volume in reducing this complication [30].

A very recent systematic review and meta-analysis based on the results of a large number of patients, evaluated a total of 19 risk factors from 93 studies. The highest risk of hypoparathyroidism occurs in female thyroid cancer patients with lymph node metastasis undergoing TT combined with neck dissection. The only outcome of this extensive meta-analysis corresponding to our results was the association of parathyroid tissue removal to hypoparathyroidism [32].

Therefore, it is possible to hypothesize that the results of our study, in highlighting a certain ‘flattening’ of the outcomes among the three categories of surgeons considered, shed light on the importance of the total volume of thyroidectomies performed and the effectiveness of mentoring in positively influencing the outcome. More generally, these results could imply that a considerable number of interventions in the role of the second surgeon would tend to bridge the gap compared to more experienced surgeons.

Surprisingly, we did not find any correlation between hypocalcemia/hypoparathyroidism and hyperthyroidism, particularly Graves’ Disease. Therefore, we believe that the patients of our cohort were somehow exposed to ‘hungry bone syndrome’ as all patients underwent thyroidectomy within 12–36 months from the onset of hyperthyroidism. However, in univariate analysis, no statistically significant difference was found between euthyroid and hyperthyroid patients in terms of post-surgical hypocalcemia/hypoparathyroidism. For this reason, this variable was not analyzed further.

The data we derived on the risk of hypocalcemia can be cross-referenced with those of other studies assessing the risk of hypoparathyroidism after thyroidectomy, considering the roles of early PTH measurement 4 hours after thyroidectomy [33] and of measurement of corrected serum calcium and PTH on the first postoperative day as predictors of safe discharge [7].

This study presents several limitations: beyond its retrospective nature, it should be noted that the cohort of patients included is quite limited. This might have led to the absence of certain statistical significances, which could have been achieved with a larger number of patients. Given the strict inclusion/exclusion criteria, expanding the patient recruitment retrospectively would have been possible; however, this would have compromised the homogeneity of the examined surgeons group, due to the increased turnover and excessive uniformity of residents, and the lack of representation of the surgeon with intermediate experience. Furthermore, the short duration of the study (three years) allowed for uniformity in the main patient evaluation criteria. The sample size was also negatively affected by its significant overlap with the pandemic period, during which strict limitations were placed on the performance of non-urgent surgical procedures [34], despite the fact that these choices led to a general worsening of disease stages, particularly in neoplastic diseases [35]. Nevertheless, we believe that limiting the study to the evaluation of only TH, excluding the definitive form, allowed us to analyze a complication that has unique characteristics and causative mechanisms, which we thus aimed to emphasize.

Finally, we want to explain why we employed multivariate analysis on data that were not significant in univariate analysis. We wanted to include in the multivariate analysis variables that were close to significance and that we deemed clinically relevant (such as age and accidental removal of parathyroid tissue). The significance results obtained are not invalidated by the univariate analysis results.

## 5. Conclusions

From the analysis of our data, it appears that the only risk factors that played a role in transient hypoparathyroidism (TH) were age over 55 years and the absence of parathyroid tissue in the specimen after resection. No other risk factors, such as malignant histology or excision extended to the neck lymph nodes, appeared to be significant. In this regard, we wish to reiterate that the sample of patients who underwent either unilateral or bilateral central neck dissection is too limited to consider this specific point of our analysis to be reliable.

Additionally, the surgeon volume of activity, categorized in our study by the total volume of thyroidectomies, does not seem to have played a leading role, although it should be considered that all surgeries were performed in a context classified as “high volume” for thyroidectomy.

We want to reiterate that a single-center study like ours, although it included homogeneous data, failed to highlight significant results such as the correlation between central neck dissection and the surgeon’s expertise with hypoparathyroidism. Nevertheless, it is still appropriate to address these variables, highlighted by numerous studies on a large number of patients, with due caution. This involves safeguarding parathyroid tissue, which should be reimplanted when necessary, and providing active mentoring for surgeons in training.

Among the significant risk factors, the removal of parathyroid tissue undeniably stands out as an area where effective preventative measures can be implemented. It is noted that such an event is most often determined by the subcapsular or intrathyroidal position of one or more parathyroids. It will therefore be possible to evaluate subsequent preventative protocols, such as the use of fluorescence techniques or other procedures capable of revealing the presence of parathyroids or parts thereof in the surgical resection.

## Figures and Tables

**Table 1:** Risk factors correlated to thyroid status (histology, thyroid functional status).

Histology	No Hypoparathyroidism (%)	Transient Hypoparathyroidism (%)	Total (%)
Benign	92 (49)	28 (54)	120 (50)
Malingnancies	72 (39)	14 (27)	86 (36)
Graves	22 (12)	10 (19)	32 (14)
Total (%)	186 (100)	52 (100)	238 (100)
**Thyroid Function**	**No Hypoparathyroidism** **(%)**	**Transient Hypoparathyroidism** **(%)**	**Total** **(%)**
Euthyroidism	144 (77)	38 (73)	182 (77)
Iperthyroidism	40 (22)	13 (25)	53 (22)
Ipothyroidism	2 (1)	1 (2)	3 (1)
Total (%)	186 (100)	52 (100)	238 (100)

**Table 2:** Risk factors correlated to surgery (parathyroid removal, surgical expertise). IRP = incidentally removed parathyroid gland.

IRP	No Hypoparathyroidism (%)	Transient Hypoparathyroidism (%)	Total **(%)**
Yes	24 (13)	12 (23)	36 (15)
No	162 (87)	40 (77)	202 (85)
Total (%)	186 (100)	52 (100)	238 (100)
**Surgeon**	**No Hypoparathyroidism** **(%)**	**Transient Hypoparathyroidism** **(%)**	**Total** **(%)**
Senior	135 (73)	38 (73)	173 (73)
Intermediate expertise	28 (15)	7 (13.5)	35 (15)
Under mentoring	23 (12)	7 (13.5)	30 (12)
Total (%)	186 (100)	52 (100)	238 (100)

**Table 3:** HypoPT = hypoparathyroidism; TH = Transient Hypoparathyroidism; TT = Total Thyroidectomy; CND = Central Neck Dissection (unilateral); bilater.CND = Bilateral Central Neck Dissection; IRP = Incidentally Removed Parathyroid Glands; Under Mentor = Surgeon Under Mentoring. It should be noted that ‘No Complications’ group displayed a mean age of 55 years, whereas TH group showed a mean age of 51. This difference achieved a “marginal” significance (*p*-value = 0.055), therefore we sought to conduct a subsequent multivariate analysis.

Univariate Analysis					
Variable	No HypoPT 186	TH 52		OR (IC 95%)	*p*-Value
Age	55	51			0.055
Sex			Total	OR (IC 95%)	*p*-value
M	49	9	58	0.5864 (0.23–1.34)	0.2047
F	137	43	180		
Total	186	52	238		
Histology			Total	OR (IC 95%)	*p*-value
Benign	92	28	120		0.1851
Cancer	72	14	86		
Graves	22	10	32		
Total	186	52	238		
Autoimmunity			Total	OR (IC 95%)	*p*-value
No	123	29	152	1.55 (0.78–3.03)	0.1926
Yes	63	23	86		
Total	186	52	238		
Thyr function			Total	OR (IC 95%)	*p*-value
Euthyroidism	144	38	182		0.5675
Iperthyroidism	40	13	53		
Ipothyroidism	2	1	3		
Total	186	52	238		
Surgery			Total	OR (IC 95%)	*p*-value
TT	171	46	217		0.2619
TT	1	1	2		
TT + CND	10	2	12		
TT + bilater.CND	4	3	7		
Total	186	52	238		
IRP			Total	OR (IC 95%)	*p*-value
1	22	11 (33.3%)	33		0.141
2	2	1 (33.3%)	3		
None	162	40 (19.8%)	202		
Total	186	52	238		
Surgeon			Total	OR (IC 95%)	*p*-value
Senior	135	38 (22%)	173		0.9364
Intermediate	28	7 (20%)	35		
Under Mentor.	23	7 (23.3%)	30		
Total	186	52	238		

**Table 4:** IRP = Incidentally Removed Parathyroid Glands. The multivariate logistic model (AIC = 245.02) indicated that the absence of incidental IRP was associated with the age class > 55 years, presenting an odds ratio (OR) of 9.015 (*p*-value < 0.05). Simultaneously, the age class > 55 years exhibited protective effects against TH, demonstrating an OR of 0.085 (*p*-value < 0.01). Similarly, the absence of parathyroids removed was found to be protective against TH, with an OR of 0.208 (*p*-value < 0.01). Double asterisk (**) refers to the isolated variables found to be significant. Single asterisk (*) refers to the significance of the two variables shown in association.

Multivariate Analysis				
Variable	OR	IC (Inf) 95%	IC (Sup) 95%	*p*-Value
Sex	0.664	0.279	1.451	0.3246
Age > 55 years	0.085	0.011	0.421	<0.01 **
No IRP	0.208	0.068	0.607	<0.01 **
No IRP + age > 55 years	9.015	1.551	76.95	<0.05 *

## Data Availability

Data are contained within the article.
